# Duodenal Ampulla Neuroendocrine Tumor with GISTs of the Proximal Jejunum: A Case Report

**DOI:** 10.3390/ijms231810351

**Published:** 2022-09-08

**Authors:** Georgiana Anca Nagy, Maria Adriana Neag, Radu Drasovean, Doinita Crisan, Romeo Ioan Chira

**Affiliations:** 1 Department of Internal Medicine, Iuliu Hatieganu University of Medicine and Pharmacy, 400006 Cluj-Napoca, Romania; 2 Department of Gastroenterology, Emergency Clinical County Hospital, 400006 Cluj-Napoca, Romania; 3 Department of Pharmacology, Toxicology and Clinical Pharmacology, Iuliu Hatieganu University of Medicine and Pharmacy, 400337 Cluj-Napoca, Romania; 4 Department of Surgery, Iuliu Hatieganu University of Medicine and Pharmacy, 400006 Cluj-Napoca, Romania; 5 Department of Pathology, Iuliu Hatieganu University of Medicine and Pharmacy, 400006 Cluj-Napoca, Romania

**Keywords:** neuroendocrine tumors, NEN, duodenal ampulla, gastrointestinal stromal tumors, GIST

## Abstract

Neuroendocrine tumors (NEN) are a type of heterogenous, slow-growing tumors, that only in about half of the cases can be found in the gastrointestinal tract. Half of these is in the small intestine. The ampullary NENs are rare, accounting for less than 1% of gastroenteropancreatic NENs. Gastrointestinal stromal tumors (GIST) are a more common type of tumors of the gastrointestinal tract that consist of pacemaker cells. The occurrence of both tumors simultaneously is rare, but in patients with neurofibromatosis type 1, the co-existence of NEN and GIST is more often. Here we report a case of simultaneous occurrence of a well-differentiated NEN and a GIST in a patient without neurofibromatosis. Also, we provide a short review of the current knowledge and treatment strategies regarding these tumors.

## 1. Introduction

The expanding use of upper gastrointestinal endoscopy in the last decade has led to an increase in the diagnosis of duodenal neuroendocrine tumors (NEN) [1]. NENs are heterogenous tumors that are found in 55% of cases in the gastrointestinal tract and in 25% in the bronchopulmonary tract. About half of the gastrointestinal NENs are in the small intestine and are a type of slow-growing tumor [2]. Depending on the secretion of hormone peptides some of them are functional, while others are non-functional. Even though NENs of the digestive system seem to have common morphological features, the behavior of the tumors can be unpredictable [3]. The ampullary NENs are rare gastroenteropancreatic NENs, accounting for less than 1% of these tumors. Most NENs occur in the first two segments of the duodenum, while those located at the ampulla of Vater are considered distinct entities because they are similar to pancreatic tumors [4]. The available data about ampullary NENs are limited to a few case-reports [5]. The determination of chromogranin A (CgA), neuron-specific-enolase (NSE) and 5-hydroxyindoleacetic acid (5-HIAA) in serum can be used for NEN diagnostics. CgA represents a glycoprotein secreted by different NENs, but in patients with severe comorbidities, such as renal failure or hypertension, or in those undergoing treatment with proton pump inhibitors, CgA levels may also be elevated [6]. Therefore, CgA is used for treatment monitoring rather than for NEN diagnostic [2].

GIST can develop anywhere in the gastrointestinal tract, from the esophagus to the rectum, with a higher incidence in the stomach (60%) and lower incidence in the duodenum and rectum (5%) [7]. The prevalence of GIST is unknown, and this type of tumor is usually discovered incidentally. GISTs express cluster of differentiation [CD] 117 (CD117) antigen (c-kit) with major inferences in the diagnosis and treatment [8]. For the diagnosis of GIST that does not express c-kit (approximately 5%), the discovered on gastrointestinal stromal tumors protein 1 (DOG1) immunohistochemistry study may be useful [9,10].

The simultaneous existence of an NEN of the duodenal ampulla and a GIST of the proximal jejunum is rare, with some cases of co-occurrence of these types of tumors being reported in patients with neurofibromatosis type 1 (NF1) [11]. Thus, we present a case of synchronous occurrence of a well-differentiated NEN of the duodenal ampulla and GISTs of the proximal jejunum, in a patient without NF1.

## 2. Case Report

A 48-year-old Caucasian patient was referred to our service for an echoendoscopy to elucidate the nature of a jejunal tumor formation shown on CT in another clinic. A subepithelial tumor of the small intestine was suspected in this patient.

The patient complained of pain in the left hypochondrium, bloating and weakness. The symptoms started three years previously and were exacerbated by exertion or orthostatism. Initial laboratory findings showed unusual high levels of gamma-glutamyl transferase (GGT) (53 U/L), elevated serum C-reactive protein levels (0.55 mg/dL), high neutrophils count (79.5%), low lymphocyte count (15.1%), elevated number of leukocytes and low level of serum iron (55 mcg/dL). CgA and neuron-specific enolase were normal.

**Figure 1:**
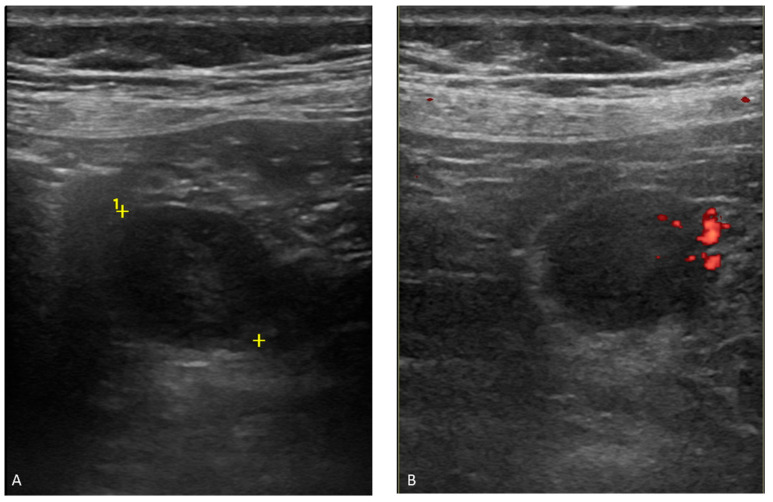
Abdominal ultrasound (US): (**A**) US mode; and (**B**) color Doppler. Hypoechoic area (2.3 cm), well delimited, at the jejunal level. Hypoechogenic tumoral mass, well delimited and with vascular pedicle.

Abdominal ultrasound examinations showed a hypoechogenic tumoral mass, well delimited and with vascular pedicle, at the jejunal level (Figure 1).

### 2.1. Abdominal and Pelvic CT with Contrast

**Figure 2:**
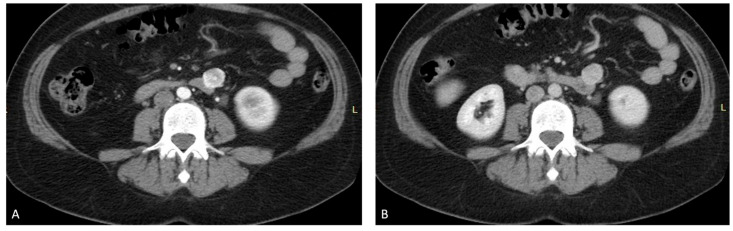
Abdominal CT with contrast: (**A**) arterial time; and (**B**) venous time. Welldefined tumor with necrotic core.

The CT of the abdomen and pelvis identified a tissue node (21 mm AP/22 mm LL/24 mm CC) in the proximal jejunum, near the Treitz angle and duodenum, that was inhomogeneous, with necrotic core and ill-defined margins. The appearance suggested a possible gastrointestinal stromal tumor (GIST). No lumbar, aortic, or iliac lymphadenopathy was observed (Figure 2).

### 2.2. Upper Enteroscopy

**Figure 3:**
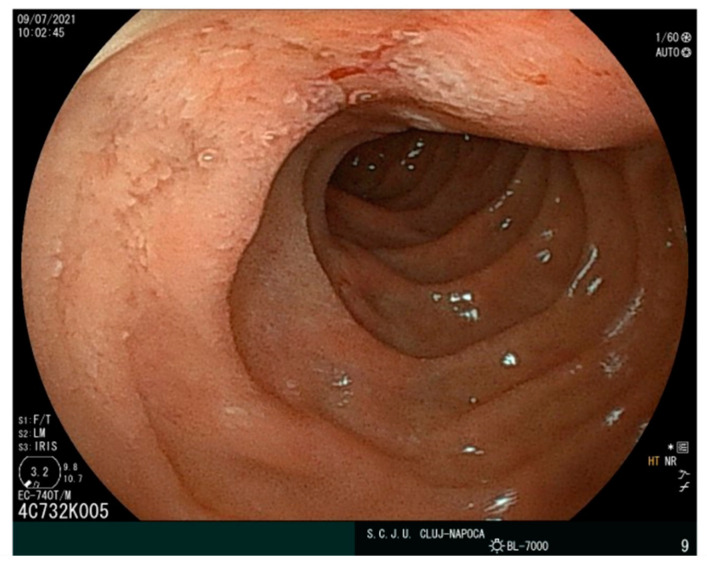
Ulceration adjacent to Vater’s ampulla, covered by fibrin.

The upper GI endoscopy showed a periampullary ulceration but no subepithelial jejunal lesions were observed intraluminal. The duodenal papilla at the second portion of the duodenum (D2) was normal. A superficial ulceration coated with fibrin, 12 mm in diameter, adjacent to the papilla was observed. Tissue samples (biopsy) were taken for histopathological examination. The other parts of the duodenum and proximal jejunum showed no changes in the mucosa or other subepithelial tumors (Figure 3).

### 2.3. Endoscopic Ultrasound (EUS)

**Figure 4:**
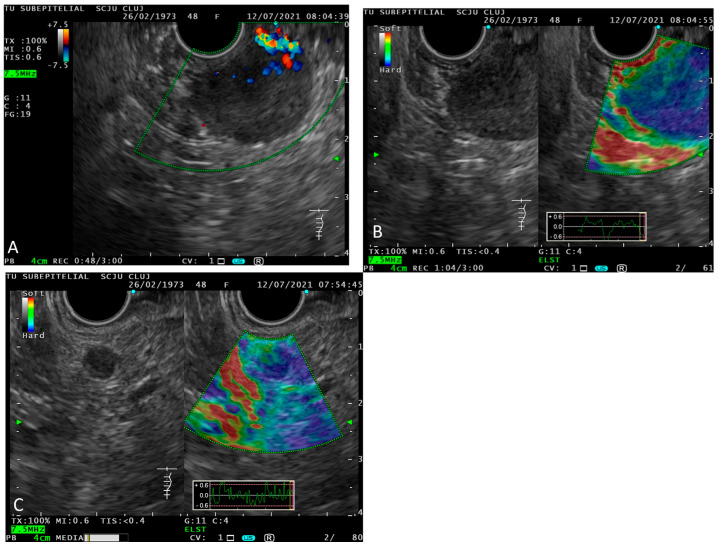
EUS periampular tumor in US, Doppler-US and elastography showing the vascular pedicle and increased rigidity of the tumoral mass (**A**,**B**); EUS periduodenal lymph node: image in (**B**) mode and elastography showing increased nodule rigidity (**C**).

Endoscopic ultrasound identified a 17 mm periampullary tumor. Doppler-US and elastography revealed the central vascularization of the tumor and its rigidity. EUS-FNA (fine needle biopsy) was performed, and two samples were taken. In addition, we noticed an inhomogeneous, 6 mm spherical mesenteric ganglion in the vicinity of the tumor (Figure 4).

**Figure 5:**
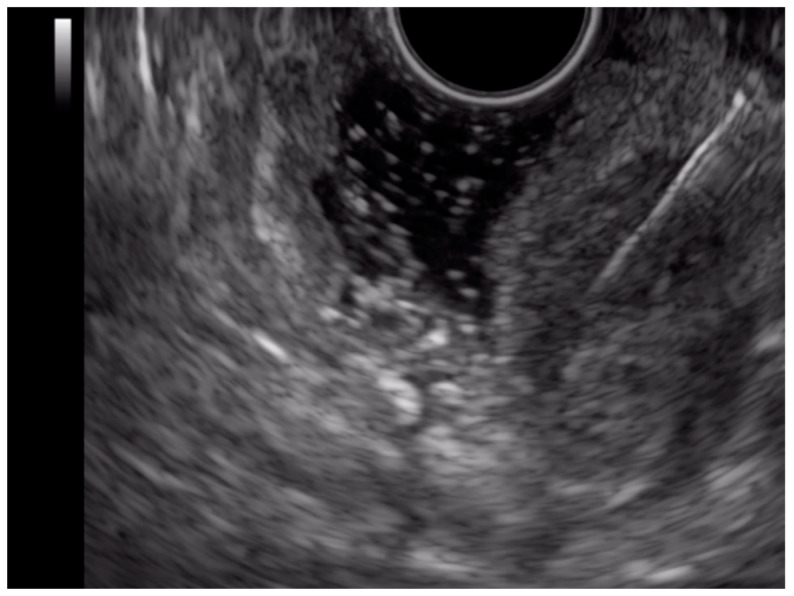
EUS jejunal tumor with FNA biopsy.

In the proximal jejunal we observed another subepithelial tumoral mass of 2.3 cm, rigid and well vascularized (Figure 5). EUS-FNA was performed, and one sample was taken.

EUS results concluded that the proximal subepithelial, periampullary and jejunal tumors were most likely NENs due to their appearance, vascularity and elastographic features.

A subepithelial tumor was observed in the proximal jejunum and a biopsy was performed. Another tumor was observed in the duodenal papilla and a biopsy was performed.

### 2.4. Histopathological Exam

Upon microscopic examination, a proliferation of neoplastic medium-sized cells with salt-and-pepper nuclei, organized in nests and trabeculae was observed. Mitotic activity was estimated as 2–3 mitoses/2 mm^2^. Lymphatic tumor emboli and perineural invasion were present, but no venous invasion. With immunohistochemistry, the tumor cells stained positive for Synaptophysin, Chromogranin A and Cytokeratin7; the KI-67 proliferative index was assessed at 2%. Those findings were consistent with a neuroendocrine tumor, G1. The tumor cells infiltrated the submucosa and the muscularis propria of the duodenum, extending into the peripancreatic tissues, with minimal invasion of the pancreatic acini. Duodenal mucosa covering the tumor was ulcerated.

Conclusion: The histological aspect correlated with immunohistochemistry argues for a well-differentiated NEN located in the duodenal mucosa.

Diagnostic: Periampullary and proximal jejunal subepithelial NENs.

After the patient was informed regarding the treatment options (surgical treatment or “watch and wait”), she decided on surgery with excision of intestinal tumors and gastrojejunal anastomosis, Roux-en-Y anastomosis (R1 resection).

Other intestinal lesions that had not been observed before were identified (three with a size of 5 mm at 20 cm, 50 cm, and 100 cm Treitz angle). Mesenteric lymphadenopathy has also been observed. On palpation, an intraluminal tumor and periduodenal lymphadenopathy were identified.

**Figure 6:**
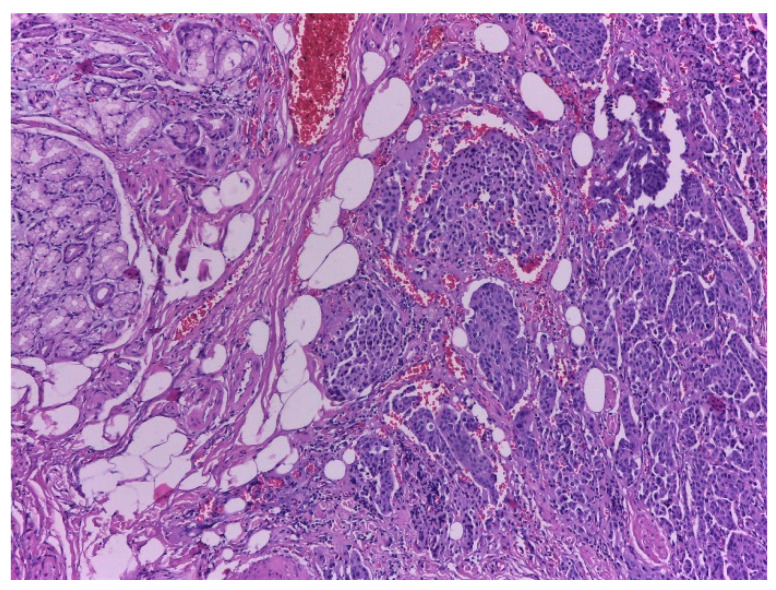
Well differentiated NEN located in the duodenal wall in Hematoxylin and eosin stain, ×100.

**Figure 7:**
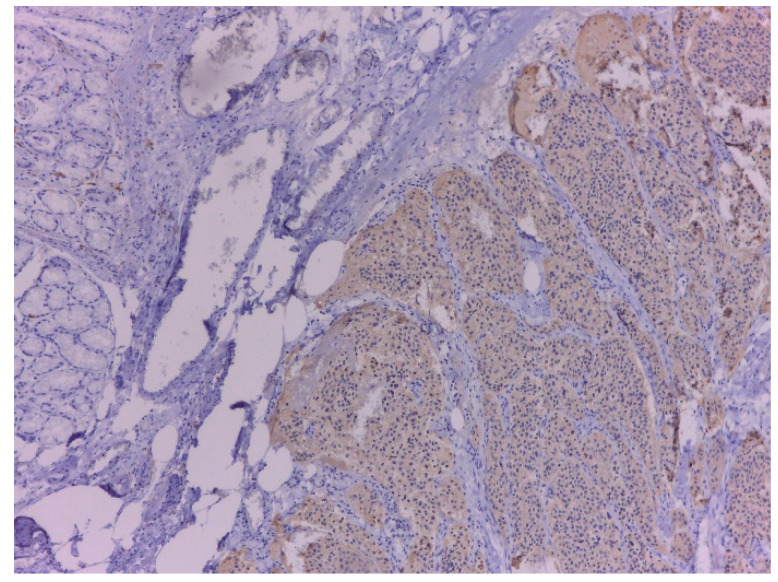
Immunohistochemical staining tumor cells of NEN are positive (stained brown) for Synapthophysin. On the left one can see the duodenal glands that are negative, ×100.

**Figure 8:**
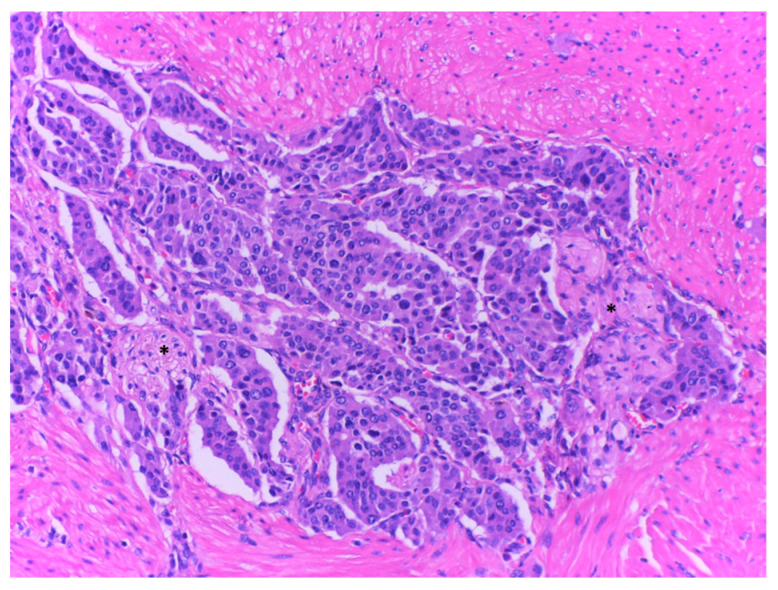
NEN infiltrating duodenal muscularis propria. Perineural infiltration is seen (*) in Hematoxylin and eosin stain, ×200.

**Figure 9:**
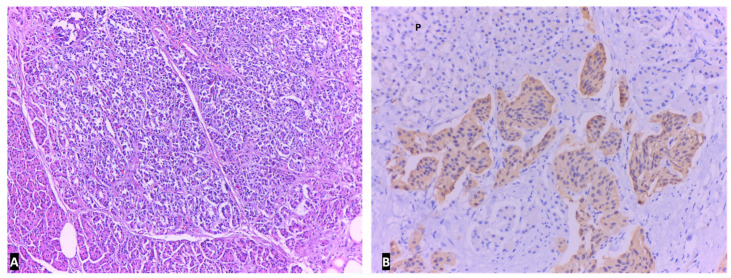
NEN infiltrating the pancreas (normal acini are seen in the lower left corner) in Hematoxylin and eosin stain, ×100: (**A**) NEN infiltrating the pancreas (P); Nests of tumor cells with bland nuclei without nucleoli. Immunohistochemical staining for Cromogranin A, ×400 (**B**).

**Figure 10:**
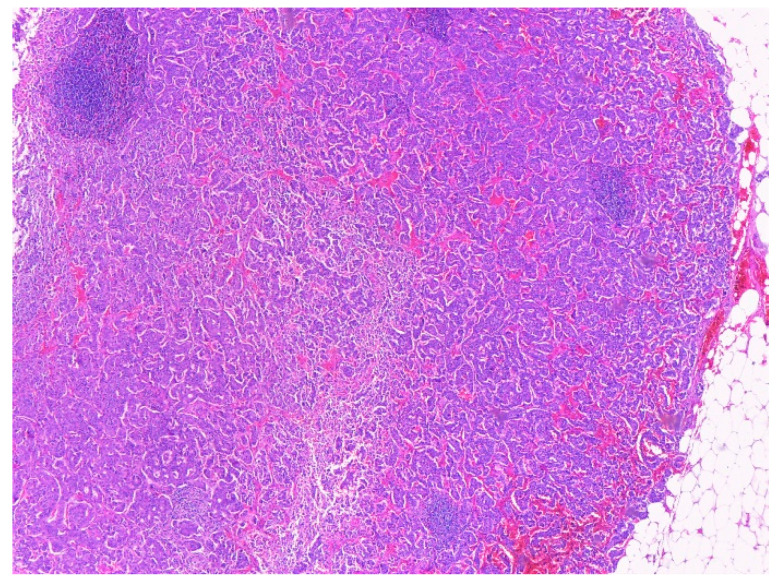
Lymph node metastasis of NEN, in Hematoxylin and eosin stain, ×40.

**Figure 11:**
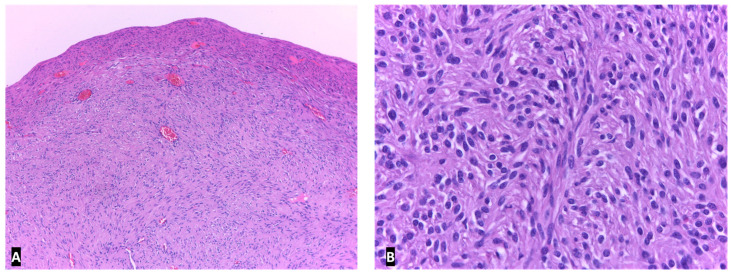
GIST of jejunum. Interlacing fascicles of monomorphus spindled cells, in Hematoxylin and eosin stain, ×40 (**A**) and ×400 (**B**).

**Figure 12:**
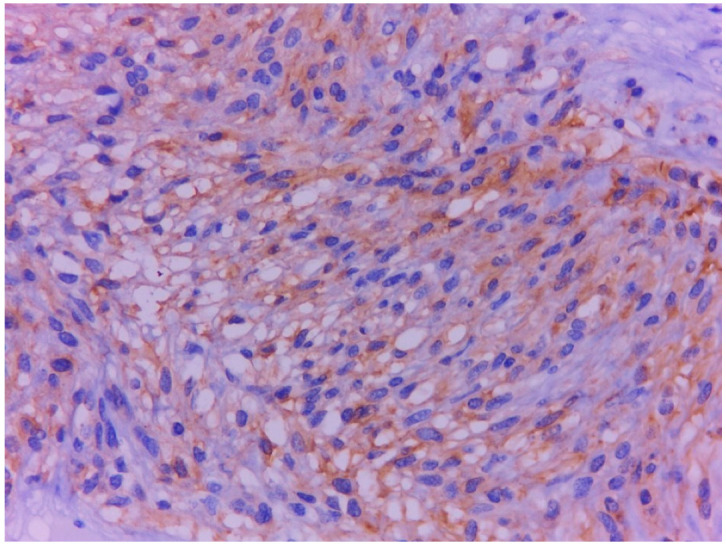
GIST tumor cells show membrane positivity for DOG1 in immunohistochemical staining, ×400.

Tests for histopathological examination revealed:

- a duodenal NEN, well delimited, with a lymph node metastasis pT3N1L1V0Pn1R1;
- four jejunal GISTs, three low-grade fusocelular and one low-grade mixed GIST (Figure 6, Figure 7, Figure 8, Figure 9, Figure 10, Figure 11 and Figure 12).

The characteristics of the NEN and GISTs samples examined are shown in Table 1.

Final diagnostic: Periduodenal NEN, well delimited pT3N1L1V0Pn1R1. Jejunal GIST three low-grade fusocelular and one low-grade mixed.

The postoperative evolution, both immediate and distant, was favorable, without complications and with the resolution of symptoms. Remote evolution depends on the incomplete resection of the periampullary NEN. The follow-up period was at nine months. At that time, no recurrence was determined by CT, PET-CT, endoscopy and echoendoscopy. Two periduodenal nodes were identified at CT, but the biopsy demonstrated their inflammatory origin.

## 3. Discussion

Our patient was 48 years old, and the symptoms started three years previously. The age for the occurrence of GIST is lower comparative to the average age previously identified for this type of tumor. A systematic review that collected data over 14 years highlighted several GIST features: the predominant age was 60 years, over 80% had symptoms, and the most common location was in the stomach and small intestine [12]. A similar average age (around 60 years) was also reported in the literature for patients diagnosed with ampullary NEN [5].

Bleeding is the most common symptom in patients with GIST [13] because of the abundant network of blood vessels in the submucosal layer of the duodenum [14]. Our patient did not present with this symptom and hemoglobin and hematocrit levels were normal. However, a slight decrease in iron serum levels was observed. For duodenal NENs the onset is insidious, and the symptoms are absent or few and nonspecific. Over time, as the disease progresses, general and nonspecific digestive symptoms (abdominal pain, bleeding, anemia, fatigue) may occur [15].

For the diagnosis of gastro-duodenal NEN and GIST, upper digestive endoscopy and digestive echoendoscopy remain the reference methods [16], while imaging techniques (CT, MRI) have limited diagnostic value for small tumors, as in the case of our patient. Ga-PET-DOTANOC would have been useful for the diagnosis of duodenal NEN, but the method is not available in our country. In the case of small subepithelial tumors (especially GIST), exploratory surgery and their surgical resection can represent a diagnostic and therapeutic strategy. The surgical intervention was performed in our case and the small intestine (duodenum, jejunum, and ileum) was carefully explored by palpation to identify small NEN or GIST (those identified by imaging methods or those not identified by them). Careful exploration of the small intestine is a very important part of the intervention because small tumors in the small intestine or metastases in the mesentery can escape from imaging detection, but can be identified directly [17].

EUS-FNA was a useful examination in the present case since we obtained adequate samples for histopathological examination. Thus, a preliminary diagnosis was established before surgery. EUS-FNA is recognized as an important method that plays an important role in the diagnosis and classification of GISTs and its combination with specific biomarkers can lead to tumor differentiation [18].

For our patient, the type of NEN was supported by histopathological examination, immunohistochemistry and positive NEN markers such as Syp and CgA. In addition to CgA and Syp, other first-generation neuroendocrine markers such as differentiation cluster 56 (CD56) and neuron-specific enolase (NSE) or second-generation neuroendocrine markers such as ISL1, INSM1 or Secretagogin, can be used for NEN diagnosis [19].

Syp and CgA are traditional markers for NEN and gold standard in endocrine pathology, with Syp being more sensitive and CgA more specific. Over time, Ki67 immunohistochemistry has become essential for determining the grade of NEN [19,20]. The grade of tumor depends on the proliferative activity of the tumor which is determined by the mitotic activity and the Ki-67 index [21]. Based on these two parameters (mitotic activity and Ki67 proliferation index) 30% of tumors have a discordant grade [20]. In our case, the grade is considered intermediate because there was a discrepancy between the mitotic rate (2–3) suggestive for an intermediate grade and the ki-67 (<2%) value that indicated a low grade. According to the guidelines, it is recommended that the higher grade to be used for classification [22].

Histology and immunohistochemistry are also essential for the diagnosis and classification of GIST. The immunohistochemistry markers used for the determination of GIST are c-kit, SMA, CD34, DOG1, desmin, S100 protein etc. [7]. In our case, all tumor samples were c-kit positive, SMA negative and two out of four were DOG1 positive. DOG1 is an important biomarker for GIST, especially because this marker is sensitive and specific for both c-kit positive and negative GISTs [23].

After establishing the diagnosis (periampullary and proximal jejunal subepithelial NENs), the therapeutic management was discussed: surgical treatment vs. “watch and wait”. Medical treatment (somatostatin analogue) was excluded because the case did not meet the protocol criteria; the tumor was not poorly differentiated, and the patient showed no clinical signs of carcinoid syndrome.

Endoscopic resection of duodenal NENs is not a feasible therapeutic strategy due to the periampullary location [16]. Conservative treatment (periodic imaging surveillance) is out of the question due to the size of the tumor and the presence of peritumoral adenopathy. Consequently, surgical resection of periampullary NET and jejunal GIST was opted for.

Two surgical treatment options were considered:

1. Cephalic duodenopancreatectomy (Whipple procedure) and segmental enterectomy for jejunal tumor with gastro-jejunal, common bile duct-jejunal and Wirsung-jejunal anastomosis in Y a la Roux. This intervention was then ruled out because our patient was young, with well-differentiated NEN, low Ki-67, without dilation of the common bile duct and Wirsung duct, and DPC is a surgery with many associated complications and high mortality and morbidity rates [24];
2. Lateral duodenotomy with periduodenal NEN resection and segmental resection of the small intestine and duodeno-jejunal anastomosis. This intervention is less radical, but with an increased risk of acute pancreatitis and of incomplete NEN resection. However, this technique is more opportune because of the pancreatic tissue is preserved and it is a low risk for the occurrence of postoperative pancreatic fistula. Due to these advantages and because this technique is recommended for curative purposes for patients without distant metastases [25], locoregional resection surgery was performed in our patient.

For patients with duodenal GIST, surgery with or without pharmacological treatment should be considered. An optimal standard surgical procedure for these cases is not yet defined due to the anatomical features of the site [26]. Regarding the surgical procedure for GIST, a study that included 300 patients with duodenal GIST showed that the type of resection (limited resection or pancreaticoduodenectomy) did not influence the prognosis. It is important to note that the surgical strategy was established based on the location and size of the tumor [27]. However, a meta-analysis concluded that limited resection is the procedure of choice for duodenal GIST whenever possible [28].

## 4. Conclusions

Although both NENs and GISTs are rare tumors and their simultaneous existence is very rare, these clinical entities should be considered in the diagnostic approach of a patient with nonspecific digestive symptoms, as they require prompt specific treatment.

Duodenal GISTs may be omitted or confused with other types of tumors (e.g., NEN) based on imaging criteria.

## Figures and Tables

**Table 1:** Characteristics of the NEN and GISTs samples examined.

Type of Tumor	NEN	GIST			
Sample	Sample	Sample 1	Sample 2	Sample 3	Sample 4
Aspect	Tumor proliferation with cells containing round, oval, pleomorphic nuclei, and fine granular chromatin. Proliferation covered the entire section and caused partial ulceration of the mucosa that crosses muscular propria layer and extends into the peripancreatic adipose tissue with discrete infiltration of pancreatic acini. Infiltration was clearer after synaptophysin labeling.	Tumor proliferation with atypical cells, fasciculate disposition, elongated nuclei, discrete nucleoli, and pale eosinophilic cytoplasm. The apparent origin of the tumor is muscularis propria	Small intestine sample with tumor proliferation, composed of cells with fascicular disposition, elongated and non- pleomorphic nuclei, pale eosinophilic cytoplasm, rare epithelioid cells. The apparent origin of the tumor is muscularis propria	Sample muscle proliferation with tumor proliferation composed of cells without atypia, fascicular disposition, elongated nuclei, discrete nuclei, and pale eosinophilic cytoplasm	Sample muscle proliferation with tumor proliferation composed of cells without atypia, fascicular disposition, elongated nuclei, discrete nuclei, and pale eosinophilic cytoplasm
Tumor cells	CgA-positive SPY-positive Ki67-reduced, maximum 2%	c-kit-positive DOG1 positive SMA-negative	c-kit-positive DOG1 positive SMA-negative CgA-negative	c-kit-positive SMA-negative	c-kit-positive SMA-negative

## Data Availability

Not applicable.

## References

[1] Massironi S., Campana D., Partelli S., Panzuto F., Rossi R.E., Faggiano A., Brighi N., Falconi M., Rinzivillo M., Fave G.D. (2018). Heterogeneity of Duodenal Neuroendocrine Tumors: An Italian Multi-center Experience. Ann. Surg. Oncol..

[2] Ahmed M. (2020). Recent advances in the management of gastrointestinal stromal tumor. World J. Clin. Cases.

[3] Yang Z., Cloyd J.M., Pawlik T.M. (2021). Pathology of Neuroendocrine Neoplasms in the Digestive System. Neuroendocrine Tumors: Surgical Evaluation and Management.

[4] Rossi R.E., Rausa E., Cavalcoli F., Conte D., Massironi S. (2018). Duodenal neuroendocrine neoplasms: A still poorly recognized clinical entity. Scand. J. Gastroenterol..

[5] Ruff S.M., Standring O., Wu G., Levy A., Anantha S., Newman E., Karpeh M.S., Nealon W., Deutsch G.B., Weiss M.J. (2021). Ampullary Neuroendocrine Tumors: Insight into a Rare Histology. Ann. Surg. Oncol..

[6] Scott A.T., Howe J. (2018). Management of Small Bowel Neuroendocrine Tumors. J. Oncol. Pract..

[7] Hirota S. (2018). Differential diagnosis of gastrointestinal stromal tumor by histopathology and immunohistochemistry. Transl. Gastroenterol. Hepatol..

[8] Parab T.M., DeRogatis M.J., Boaz A.M., Grasso S.A., Issack P.S., Duarte D.A., Urayeneza O., Vahdat S., Qiao J.-H., Hinika G.S. (2018). Gastrointestinal stromal tumors: A comprehensive review. J. Gastrointest. Oncol..

[9] Mei L., Du W., Idowu M., Von Mehren M., Boikos S.A. (2018). Advances and Challenges on Management of Gastrointestinal Stromal Tumors. Front. Oncol..

[10] Pyuza J.J., Shao E.R., Bosco K., Lodhia J., Mremi A. (2021). An incidental finding of duodenal GIST in a patient with penetrating abdominal trauma: A case report. Int. J. Surg. Case Rep..

[11] Park E.K., Kim H.J., Lee Y.H., Koh Y.S., Hur Y.H., Cho C.K. (2019). Synchronous Gastrointestinal Stromal Tumor and Ampullary Neuroendocrine Tumor in Association with Neurofibromatosis Type 1: A Report of Three Cases. Korean J. Gastroenterol..

[12] Søreide K., Sandvik O.M., Søreide J.A., Giljaca V., Jureckova A., Bulusu V.R. (2016). Global epidemiology of gastrointestinal stromal tumours (GIST): A systematic review of population-based cohort studies. Cancer Epidemiol..

[13] Chan K.P. (2018). What’s the Mass? The Gist of Point-of-care Ultrasound in Gastrointestinal Stromal Tumors. Clin. Pract. Cases Emerg. Med..

[14] Futo Y., Saito S., Miyato H., Sadatomo A., Kaneko Y., Kono Y., Matsubara D., Horie H., Lefor A.K., Sata N. (2018). Duodenal gastrointestinal stromal tumors appear similar to pancreatic neuroendocrine tumors: A case report. Int. J. Surg. Case Rep..

[15] Wang X., Wu Y., Cao X., Zhang X., Cheng Y., Kong L. (2021). Duodenal neuroendocrine tumor. Medicine.

[16] Delle Fave G., O’Toole D., Sundin A., Taal B., Ferolla P., Ramage J., Ferone D., Ito T., Weber W., Zheng-Pei Z. (2016). ENETS Consensus Guidelines Update for Gastroduodenal Neuroendocrine Neoplasms. Neuroendocrinology.

[17] Tran C., Sherman S.K., Howe J.R. (2020). Small Bowel Neuroendocrine Tumors. Curr. Probl. Surg..

[18] Gilani S.M., Muniraj T., Aslanian H.R., Cai G. (2020). Endoscopic ultrasound-guided fine needle aspiration cytology diagnosis of upper gastrointestinal tract mesenchymal tumors: Impact of rapid onsite evaluation and correlation with histopathologic follow-up. Diagn. Cytopathol..

[19] Juhlin C.C. (2021). Second-Generation Neuroendocrine Immunohistochemical Markers: Reflections from Clinical Implementation. Biology.

[20] Bellizzi A.M. (2019). Immunohistochemistry in the diagnosis and classification of neuroendocrine neoplasms: What can brown do for you?. Hum. Pathol..

[21] Nagtegaal I.D., Odze R.D., Klimstra D., Paradis V., Rugge M., Schirmacher P., Washington K.M., Carneiro F., Cree I.A., The WHO Classification of Tumours Editorial Board (2020). The 2019 WHO classification of tumours of the digestive system. Histopathology.

[22] Shah M.H., Goldner W.S., Benson A.B., Bergsland E., Blaszkowsky L.S., Brock P., Chan J., Das S., Dickson P.V., Fanta P. (2021). Neuroendocrine and Adrenal Tumors, Version 2.2021, NCCN Clinical Practice Guidelines in Oncology. J. Natl. Compr. Cancer Netw..

[23] Karakas C., Christensen P., Baek D., Jung M., Ro J.Y. (2018). Dedifferentiated gastrointestinal stromal tumor: Recent advances. Ann. Diagn. Pathol..

[24] D’Cruz J.R., Misra S., Shamsudeen S. (2022). Pancreaticoduodenectomy. StatPearls.

[25] Daskalakis K., Tsolakis A.V. (2018). Upfront surgery of small intestinal neuroendocrine tumors. Time to reconsider?. World J. Gastroenterol..

[26] Lee S.Y., Goh B.K.P., Sadot E., Rajeev R., Balachandran V.P., Gönen M., Kingham T.P., Allen P.J., D’Angelica M.I., Jarnagin W.R. (2016). Surgical Strategy and Outcomes in Duodenal Gastrointestinal Stromal Tumor. Ann. Surg. Oncol..

[27] Liu Z., Zheng G., Liu J., Liu S., Xu G., Wang Q., Guo M., Lian X., Zhang H., Feng F. (2018). Clinicopathological features, surgical strategy and prognosis of duodenal gastrointestinal stromal tumors: A series of 300 patients. BMC Cancer.

[28] Chok A.-Y., Koh Y.-X., Ow M.Y.L., Allen J.C., Goh B.K.P. (2014). A Systematic Review and Meta-analysis Comparing Pancreaticoduodenectomy Versus Limited Resection for Duodenal Gastrointestinal Stromal Tumors. Ann. Surg. Oncol..

